# Supplementary material for: Comprehensive Analysis of CXCR4, JUNB, and PD-L1 Expression in Circulating Tumor Cells (CTCs) from Prostate Cancer Patients
Source: Cells. 2024 May 3;13(9):782. doi: 10.3390/cells13090782 (PMC11083423; doi:10.3390/cells13090782)
Supplement: Supplementary file 1 [file cells-13-00782-s001.zip › cells-2949848-supplementary.pdf]

Supplementary Figure S1

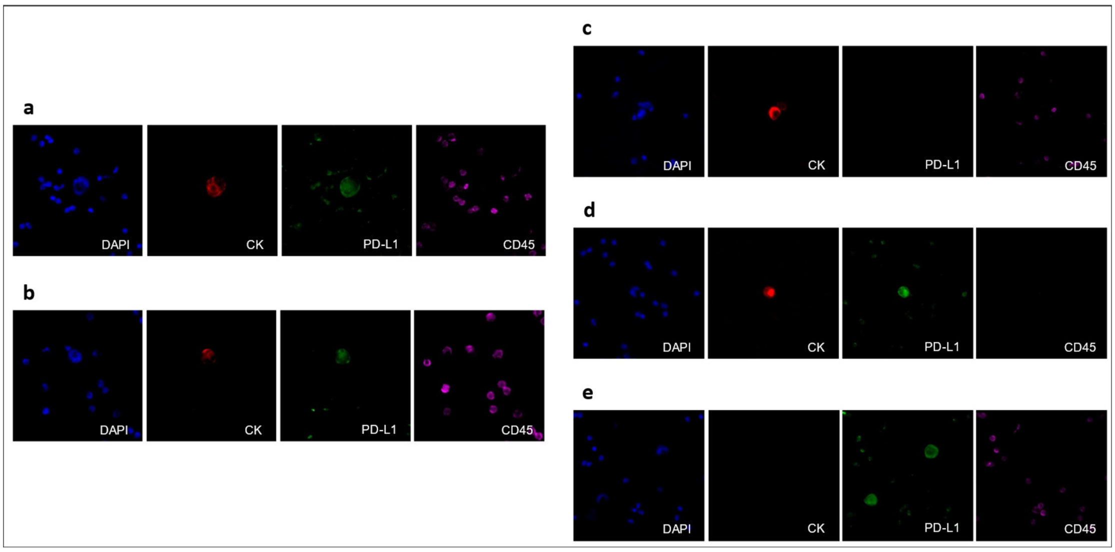

Supplementary Figure S2

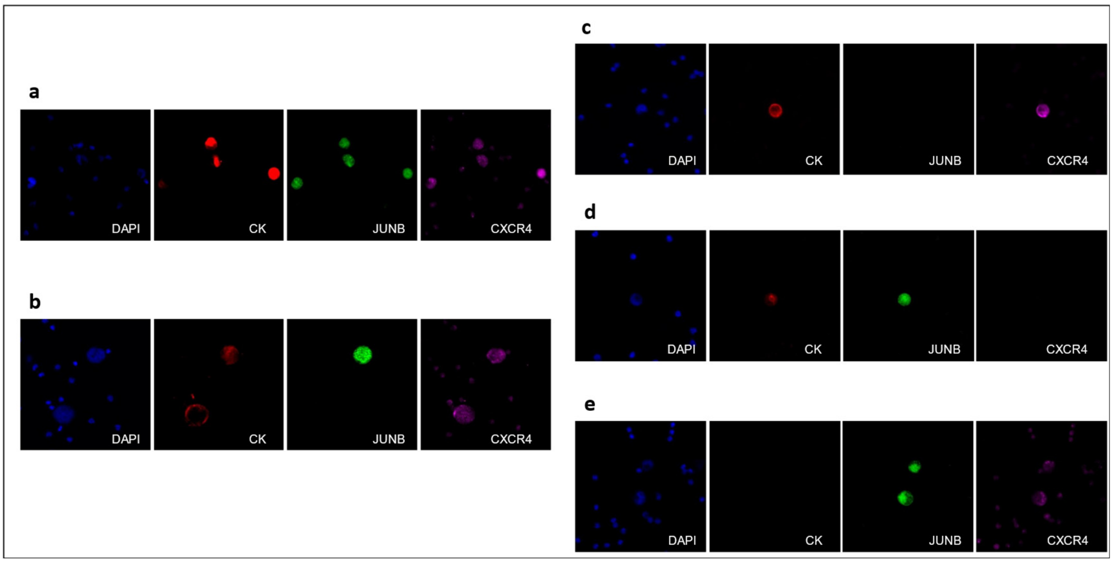

Supplementary Figure S3

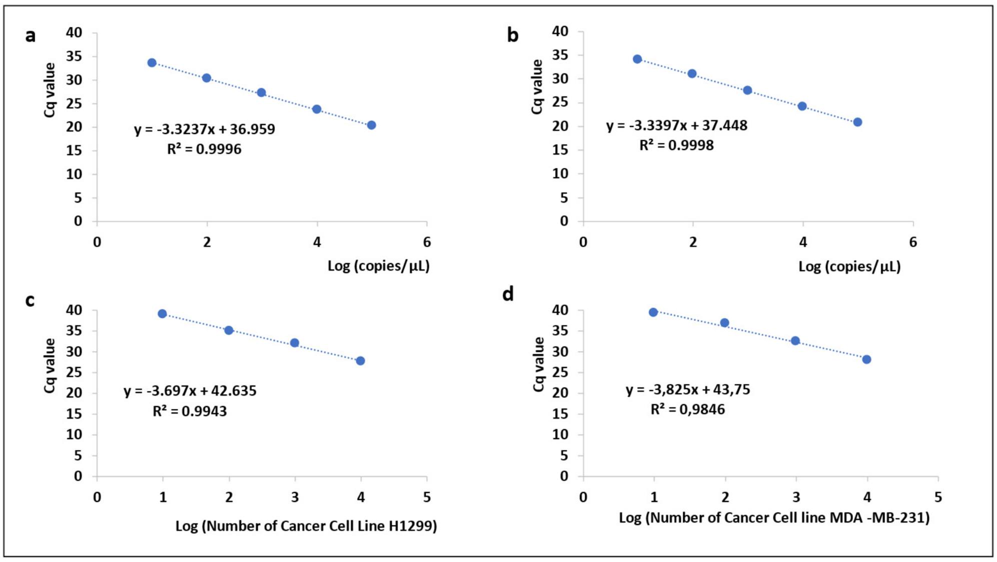

Supplementary Figure S4

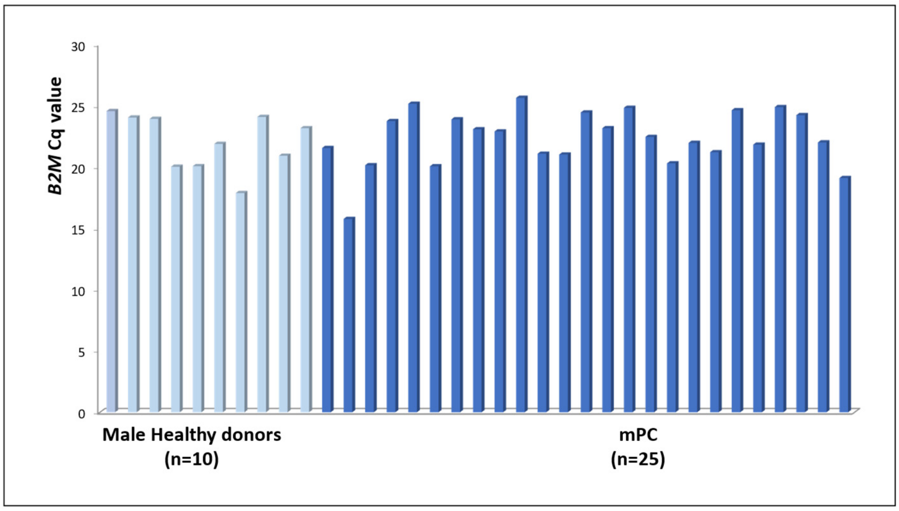

Supplementary Figure S5a

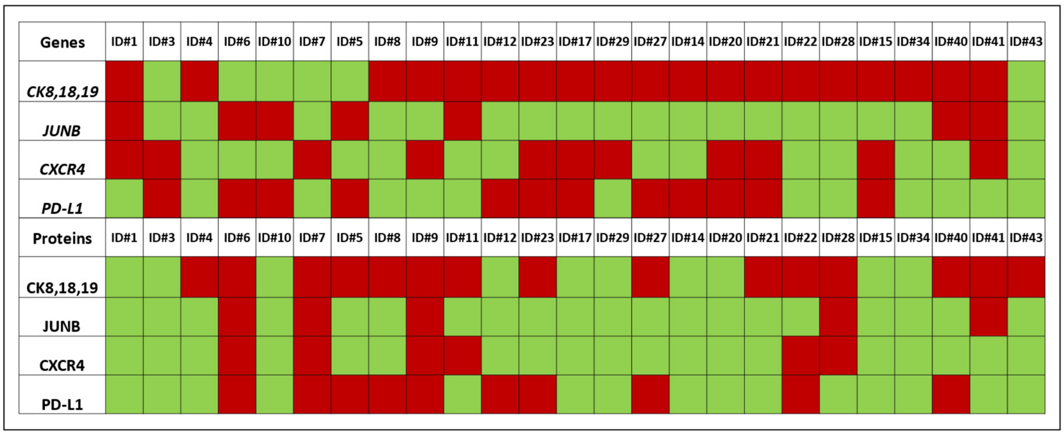

Supplementary Figure S5b

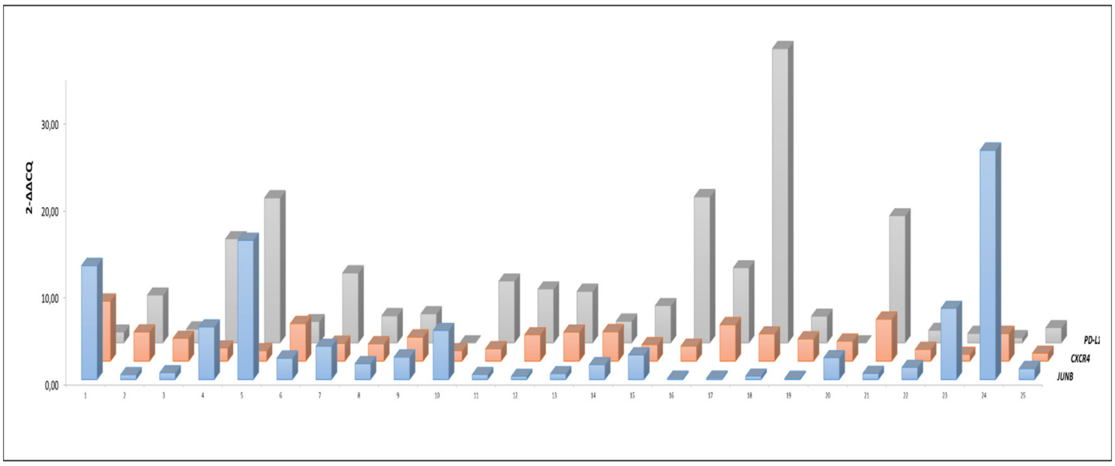

Table S1. Patients' characteristics enrolled in Ficoll density isolation (48 patients).

Variable	Sub-categories<br>n (%)
**Age**	
≥ 75	20 (41.7 %)
< 75	13 (27.1 %)
Unknown	15 (31.2 %)

**Gleason score, n (%)**	
≥ 8	24 (50 %)
< 8	14 (29.2 %)
Unknown	10 (20.8 %)
**Mean PSA (Range), (ng/mL)**	89.9 (1.6-342)
**Smoking Consumption**	
Yes	13 (27.1 %)
No	27 (56.2 %)
Unknown	8 (16.7 %)
**Disease stage**	
Metastatic	48 (100.0 %)
**Metastasis locations**	
Bone metastasis	19 (39.6 %)
Lymph node metastasis	12 (25.0 %)
Lung metastasis	4 (8.33 %)
Bone marrow metastasis	1 (2.1 %)
Kidney metastasis	1 (2.1 %)
Unknown	11 (22.9 %)
**Chemotherapy**	
Baseline	38 (79.2 %)
After 1st line	10 (20.8 %)

Table S2. Patients' characteristics enrolled in mRNA analysis (25 patients).

Variable	Sub-categories n (%)
**Age**	
≥ 75	8 (32.0 %)
< 75	8 (32.0 %)
Unknown	9 (36.0 %)
**Gleason score, n (%)**	
≥ 8	12 (48.0 %)

< 8	7 (28.0 %)
Unknown	6 (24.0 %)
**Mean PSA (Range), (ng/mL)**	65.4 (2-251)
**Smoking Consumption**	
Yes	4 (16.0 %)
No	16 (64.0 %)
Unknown	5 (20.0 %)
**Disease stage**	
Metastatic	25 (100.0 %)
**Metastasis locations**	
Bone metastasis	8 (32.0 %)
Lymph node metastasis	9 (36.0 %)
Lung metastasis	1 (4.0 %)
Bone marrow metastasis	1 (4.0 %)
Kidney metastasis	1 (4.0 %)
Unknown	5 (20.0 %)
**Chemotherapy**	
Baseline	25 (100.0 %)
After 1st line	0 (0 %)

Table S3. Patients' characteristics enrolled in ISET isolation (17 patients).

**Variable**	**Sub-categories n (%)**
**Age**	
≥ 75	7 (41.2 %)
< 75	8 (47.0 %)
Unknown	2 (11.8 %)
**Gleason score, n (%)**	
≥ 8	6 (35.3 %)
< 8	5 (29.4 %)
Unknown	6 (35.3 %)

**Mean PSA (Range), (ng/mL)**	95.2 (7.9-251)
**Smoking Consumption**	
Yes	6 (35.3 %)
No	7 (41.2 %)
Unknown	4 (23.5 %)
**Disease stage**	
Metastatic	17 (100.0 %)
**Metastasis locations**	
Bone metastasis	9 (52.9 %)
Lymph node metastasis	6 (35.3 %)
Lung metastasis	2 (11.8%)
Bone marrow metastasis	0 (0 %)
Kidney metastasis	0 (0 %)
Unknown	0 (0 %)
**Chemotherapy**	
Baseline	17 (100.0 %)
After 1st line	0 (0 %)

Table S4. CTCs per patient after ficoll density gradient isolation and triple immunofluorescent staining CK/PD-L1/CD45.

Patient ID#number	Total CK+ cells (CTCs)	CK+/PD-L1+/CD45-	CK+/PD-L1-/CD45-
**1**	0	0	0
**2**	1	1	0
**3**	0	0	0
**4**	1	0	1
**5**	5	5	0
**6**	6	0	6
**7**	1	1	0
**8**	2	2	0
**9**	0	0	0

**10**	0	0	0
**11**	0	0	0
**12**	0	0	0
**13**	0	0	0
**14**	0	0	0
**15**	0	0	0
**16**	1	1	0
**17**	0	0	0
**18**	0	0	0
**19**	0	0	0
**20**	0	0	0
**21**	1	0	1
**22**	1	1	0
**23**	2	2	0
**24**	1	1	0
**25**	3	3	0
**26**	0	0	0
**27**	1	1	0
**28**	2	0	2
**29**	0	0	0
**30**	0	0	0
**31**	0	0	0
**32**	0	0	0
**33**	0	0	0
**34**	0	0	0
**35**	0	0	0
**36**	0	0	0
**37**	0	0	0
**38**	0	0	0
**39**	0	0	0
**40**	1	1	0
**41**	0	0	0
**42**	1	0	1
**43**	1	0	1
**44**	0	0	0
**45**	1	0	1
**46**	0	0	0

**47**	0	0	0
**48**	0	0	0

Table S5. Wilcoxon signed-rank test results for the identified phenotypes from triple immunofluorescence stainings.

	**CK+/PD-L1+/CD45- vs CK+/PD-L1-/CD45-**	**ISET_CK+/PD-L1+/CD45- vs ISET_CK+/PD-L1-/CD45-**	**CK+/CXCR4+ vs CK+/JUNB+**	**CK+/CXCR4+/JUNB+ vs CK+/CXCR4-/JUNB+**	**CK+/CXCR4+/JUNB+ vs CK+/CXCR4+/JUNB-**	**CK+/CXCR4+/JUNB+ vs CK+/CXCR4-/JUNB-**	**CK+/CXCR4+/JUNB- vs CK+/CXCR4-/JUNB+**	**CK+/CXCR4-/JUNB+ vs CK+/CXCR4-/JUNB-**
**Asymp. Sig. (2-tailed)**	**0.343**	**0.861**	**0.518**	**0.013**	**0.083**	**0.077**	**0.518**	**0.336**

Table S6. CTCs per patients after ISET isolation and triple immunofluorescent staining CK/PD-L1/CD45.

**Patient ID#number**	**Total CK+ cells (CTCs)**	**CK+/PD-L1+/CD45-**	**CK+/PD-L1-/CD45-**
**5**	2	1	1
**6**	1	1	0
**7**	3	1	2
**8**	97	0	97
**9**	1	1	0
**10**	0	0	0
**11**	0	0	0
**12**	1	1	0
**13**	0	0	0
**14**	0	0	0
**16**	0	0	0
**22**	0	0	0
**23**	14	8	6
**24**	1	0	1
**26**	0	0	0
**36**	0	0	0
**41**	0	0	0

Table S7. CTCs per patient after ficoll density gradient isolation and triple immunofluorescent staining CK/CXCR4/JUNB.

Patient ID#number	Total CK+ cells (CTCs)	CK+/CXCR4+/JUNB+	CK+/CXCR4-/JUNB+	CK+/CXCR4+/JUNB-	CK+/CXCR4-/JUNB-
**1**	0	0	0	0	0
**2**	1	1	0	0	0
**3**	0	0	0	0	0
**4**	3	0	0	0	3
**5**	0	0	0	0	0
**6**	16	8	3	2	3
**7**	5	2	0	3	0
**8**	0	0	0	0	0
**9**	1	1	0	0	0
**10**	0	0	0	0	0
**11**	2	0	0	2	0
**12**	0	0	0	0	0
**13**	1	1	0	0	0
**14**	0	0	0	0	0
**15**	0	0	0	0	0
**16**	2	2	0	0	0
**17**	0	0	0	0	0
**18**	0	0	0	0	0
**19**	0	0	0	0	0
**20**	0	0	0	0	0
**21**	0	0	0	0	0
**22**	1	0	0	1	0
**23**	0	0	0	0	0
**24**	1	1	0	0	0
**25**	2	2	0	0	0
**26**	0	0	0	0	0
**27**	0	0	0	0	0
**28**	4	2	0	0	2
**29**	0	0	0	0	0
**30**	1	1	0	0	0
**31**	0	0	0	0	0

**32**	0	0	0	0	0
**33**	1	0	1	0	0
**34**	0	0	0	0	0
**35**	0	0	0	0	0
**36**	0	0	0	0	0
**37**	0	0	0	0	0
**38**	0	0	0	0	0
**39**	0	0	0	0	0
**40**	1	0	0	0	1
**41**	1	0	1	0	0
**42**	0	0	0	0	0
**43**	0	0	0	0	0
**44**	0	0	0	0	0
**45**	0	0	0	0	0
**46**	0	0	0	0	0
**47**	0	0	0	0	0
**48**	0	0	0	0	0
